# Supplementary material for: A set of multi-entry identification keys to African frugivorous flies (Diptera, Tephritidae)
Source: Zookeys. 2014 Jul 24;(428):97–108. doi: 10.3897/zookeys.428.7366 (PMC4143993; doi:10.3897/zookeys.428.7366)
Supplement: Supplementary material 6 — Key to Ceratitis [file zookeys-428-097-s006.zip › SF6_ZooKeys_key to Ceratitis/key/SF6_ZooKeys_key to Ceratitis/Media/Html/Ceratitis tananarivana .htm]

Ceratitis (Pterandrus) tananarivana Hancock


***Ceratitis*** **(*Pterandrus*) *tananarivana*
Hancock**

*Ceratitis* (*Pterandrus*) *tananarivana*
Hancock, 1984: 287.

Body
length: 4.71 (4.45-5.00) mm; wing length: 5.12 (5.00-5.40) mm.

Male

Head: Antenna yellowish orange, third segment sometimes
partly brown. First flagellomere three times as long as pedicel. Arista with
short rays; ventral rays slightly shorter and sparser than dorsal rays,
especially basally. Frons flat with median longitudinal groove, with
protuberance at occellar triangle; yellow, slightly golden shine in front of
ocellar triangle, continued down longitudinal groove when viewed from certain
angle; with short scattered setulae largely of same color as frons. Frontal
setae less developed. Face yellowish white to yellow. Genal seta and setulae
dark, well developed.

Thorax:
Postpronotal lobe white, with small brownish spot, sometimes weakly developed.
Scutal pattern: ground color brown, lateral margin darker; microtrichia pattern
silvery, specimen from USNM with distinct orange tinge; mixed pale and dark pilosity;
prescutellar white or yellow-white markings separate but with pale area in
between. Scapular setae dark. Scutellum yellowish white, basally without spots,
apically with three separate black spots, extending to basal 0.33. Anepisternum
on ventral half darker yellow; with very dense silvery pilosity.

Legs:
Yellow except where otherwise noted; setation typical for subgenus, mixed pale
and dark. Foreleg: femur posteriorly on dorsal half with very dense bush of
white setulae, on ventral half with shorter and more dispersed dark setulae,
ventral setae long and black; tibia flattened dorsoventrally and expanded
laterally, white, with short (and few longer) white setulae. Midleg: tibia
flattened dorsoventrally, distal 0.17 thickened, club-like; pale, thickened
part dark, silvery when viewed from certain angle; with black feathering
dorsally and ventrally; tarsi flattened, white, silvery ventrally. Hindfemur
at apical 0.25 with longer setulae dorsally and ventrally.

Wing:
banding yellowish brown. Interruption between marginal and discal bands
near vein R_1_ clear and complete; cubital band free; medial band
joined with marginal band; crossvein R-M opposite middle of discal cell. Apex
of vein R_1_ distal to level of crossvein R-M. Crossvein DM-Cu oblique
posterobasally.

Abdomen:
Tergite 3 brown except two pale gray spots posteriorly; tergite 4 with brown
band along anterior half, with median notch; tergite 5 with two yellowish brown
spots along anterior 0.66, sometimes connected medially. Tergites 2 and 4 pale
gray (not silvery) across posterior half. Male epandrium in lateral view
moderately broadened; posterior lobe of lateral surstylus short and straight.

Female

As
male except following characters: Frons with dorsal protuberance and golden
shine less developed; frontal setae well developed. Anepisternal pilosity more
dispersed. Legs normal shape and without feathering; femora in two specimens
examined variable (yellow, or more orange colored); pilosity dark; forefemur
with dark setulae. Oviscape shorter than preabdomen. Aculeus at most six times
longer than wide; tip pointed, and lateral margin slightly sinuous.

(Description
after De Meyer & Freidberg, 2006)
